# 2025 ESC/EACTS Valvular Guidelines: imaging perspective over a new treatment direction

**DOI:** 10.1093/ehjimp/qyag002

**Published:** 2026-01-08

**Authors:** Edoardo Zancanaro, Roberto Lorusso, Tommaso Hinna Danesi

**Affiliations:** Division of Cardiac Surgery, Mass General Brigham, Brigham and Women's Hospital, Harvard Medical School, 15 St Francis Street, Boston, MA, USA; Department of Cardiac Surgery, Istituto di Ricovero e Cura a Carattere Scientifico San Raffaele Hospital, Via Olgettina 69, Milano, Italy; Cardiovascular Research Institute (CARIM), University of Maastricht, room H 1, Universiteitssingel 50, 632, 6229 ER Maastricht, The Netherlands; Cardiovascular Research Institute (CARIM), University of Maastricht, room H 1, Universiteitssingel 50, 632, 6229 ER Maastricht, The Netherlands; Department of Cardiac Surgery, Istituto di Ricovero e Cura a Carattere Scientifico San Raffaele Hospital, Via Olgettina 69, Milano, Italy

The 2025 ESC/EACTS Guidelines on valvular heart disease arrive at a moment when ‘treatment direction’ is no longer a single choice between surgery and catheter therapy but a longitudinal strategy—indexed to lifetime risk, feasibility of repeat procedures, and the real (often competing) priorities of patients. In that landscape, imaging is not simply diagnostic: it is the language that allows the Heart Team to translate anatomy, haemodynamics, symptoms, comorbidity, and procedural risk into a coherent plan. The guideline update explicitly reinforces this centrality—linking Heart Team decision-making, Heart Valve Centres, and ‘advanced diagnostic imaging with more clearly defined diagnostic criteria’ as pillars of contemporary care.

What is most striking is not any single new class of recommendation, but the way the document treats imaging as the gatekeeper of ‘when’ to intervene, ‘how’ to intervene, and ‘what comes next’ after the index procedure. That perspective is worth making explicit—because for imagers, the guideline is less a list of threshold values and more an invitation to build reproducible, multimodality pathways that can support increasingly complex treatment trajectories.^1^

## First news: the headline shifts that hint at a new direction

Several early headlines changed to signal the guideline’s intent to modernize selection and timing decisions while preserving a patient-centred, multidisciplinary framework.

### Aortic stenosis: age remains important, but lifetime strategy becomes explicit

A notable ‘headline’ change is the age cut-off moving from 75 to 70 years in favour of transcatheter aortic valve intervention (TAVI) for patients with tricuspid aortic valve stenosis, as summarized by EACTS. The official guideline educational materials also visualize age (≥70 vs. <70 years) as a key discriminator in a broader matrix that includes anatomic features, concomitant disease, and lifetime management considerations (including redo options and coronary access). The message is not ‘TAVI for everyone at 70’, but rather that age now functions within a more overtly lifetime-oriented framework—one in which imaging-derived anatomy and feasibility of future procedures matter as much as operative risk at the index intervention.

### Primary mitral regurgitation: surgery remains the anchor, but timing is pushed earlier for selected patients

EACTS highlights that surgery remains the preferred choice for primary mitral regurgitation (PMR). Importantly, a new Class I recommendation for mitral valve repair surgery in ‘asymptomatic’ PMR is emphasized for patients meeting specific diagnostic criteria. This is a strong statement: it places a premium on precise imaging phenotyping and on detecting the transition from ‘severe but compensated’ to ‘severe with subtle damage’—often before symptoms become a reliable trigger.

### Atrial secondary mitral regurgitation: recognition of phenotype and a more nuanced interventional spectrum

The guidelines introduce a new Class IIa recommendation for surgery and a Class IIb recommendation for transcatheter procedures in atrial secondary mitral regurgitation (MR), as reported by EACTS. Beyond the class labels, this legitimizes atrial secondary MR as a distinct entity whose mechanisms and therapeutic targets differ from ventricular secondary MR—again making careful imaging definition essential.

### Tricuspid regurgitation: transcatheter therapy enters the guideline mainstream—conditioned by imaging of right ventricle/pulmonary hypertension

A clear new direction is visible in the tricuspid space: transcatheter tricuspid valve treatment ‘should be considered’ (Class IIa) in high-risk symptomatic severe Tricuspid Regurgitation (TR) despite optimal medical therapy, ‘in the absence of severe RV dysfunction or pre-capillary pulmonary hypertension’. This is not a blanket endorsement; it is an imaging-contingent recommendation that forces the field to standardize how we grade TR severity, quantify right-sided remodelling, and define RV ‘point-of-no-return’.

Taken together, these ‘first news’ items are unified by a common logic: earlier, more personalized intervention—provided imaging can define severity, mechanism, chamber consequences, and procedural feasibility with confidence.^1^

## The multimodality imaging role: from ‘confirm severity’ to ‘shape the pathway’

If the guideline’s therapeutic direction is towards lifetime planning, imaging must evolve from episodic reporting to an integrative decision infrastructure. That requires three practical shifts:

1. Move from single-parameter thresholds to coherent phenotypes (mechanism + haemodynamic severity + chamber consequences + myocardial damage).
2. Use the right modality at the right decision point—not as ‘extra imaging’, but as targeted problem-solving.
3. Standardize acquisition and reporting so that Heart Teams can trust longitudinal comparisons and multicentre transfers—particularly within Heart Valve Centres.

Below is a modality-by-modality view of how this plays out across modern valve care.

## Echocardiography: still first-line, now expected to be ‘integrative’

Echocardiography remains the frontline tool, but the bar has risen: an echo report increasingly needs to function as a structured clinical argument.

### Aortic stenosis and discordance

The 2025 guideline educational algorithm for suspected severe aortic Stenosis (AS) explicitly operationalizes an integrative pathway: confirm valve area criteria, exclude measurement error, use flow status and left ventricle ejection fraction, deploy dobutamine stress echocardiography when appropriate, and incorporate computed tomography (CT) aortic valve calcium scoring with sex-specific thresholds (visualized as >1200 AU in women and >2000 AU in men) to adjudicate severity. This is not merely a technical flow chart; it is a formal acknowledgement that ‘discordant AS is common’ and that multimodality adjudication is no longer optional in high-stakes decisions (e.g. an intervention at borderline gradients).

### Mitral and TR: grading severity as a Heart Team contract

The EACVI/ESC position paper on native valvular regurgitation stresses a multimodality approach that integrates quantification, valve anatomy, and chamber consequences—precisely because management decisions increasingly depend on subtle evidence of decompensation rather than symptoms alone.^2^ For mitral regurgitation, this includes careful differentiation of mechanism (primary vs. secondary, and now atrial secondary), and for TR, it implies that echo must do more than label ‘severe’: it must quantify right-sided remodelling and function in a reproducible way that can support intervention selection and timing.^2^

### Why 3-dimensional and stress echoes matter more now

As transcatheter options expand and surgery is recommended earlier in select asymptomatic PMR, the quality of anatomical definition [3-dimensional (3D) leaflet analysis, annular geometry, coaptation gap] and functional reserve assessment becomes the difference between a confident repair-first strategy and therapeutic hesitation.^2^

## Cardiac CT: anatomy, feasibility, and the ‘lifetime’ layer

CT is increasingly the modality that turns ‘a valve lesion’ into ‘a feasible treatment plan’.

### AS: calcium scoring as an adjudication tool

The guideline’s integrative AS approach explicitly includes CT calcium scoring thresholds to resolve uncertainty. This elevates CT from a planning tool to a diagnostic arbitrator—particularly relevant in low-flow/low-gradient scenarios where echo alone may be ambiguous.

### TAVI vs. surgical aortic valve replacement selection: imaging defines the non-age drivers

The guideline’s visual framework for choosing intervention mode lists anatomic features—hostile annulus/left ventricular outflow tract calcification, bicuspid anatomy, annular dimensions unsuitable for TAVI, and coronary obstruction risk—as factors favouring surgery, while transfemoral feasibility, porcelain aorta, and intact bypass grafts may favour TAVI. The significance for imagers is direct: ‘CT-derived anatomy now carries guideline-level weight in modality selection’, not just in procedural planning.

### Lifetime planning: coronary access, redo strategies, and valve-in-valve consequences

The same figure explicitly calls out lifetime management issues such as coronary obstruction risk and impaired coronary access after valve-in-valve TAVI. This is an imaging mandate: pre-procedural CT interpretation must anticipate downstream scenarios, not only immediate feasibility.

## Cardiac MR: the quantitative referee for volumes, regurgitation, and myocardial damage

Cardiac MR’s (Magnetic Resonance) strength is not replacing echo; it is resolving what echo cannot reliably quantify when decisions are close.

For regurgitation, the EACVI/ESC position paper explicitly integrates CMR into the multimodality assessment of native valve regurgitation, recognizing its ability to quantify regurgitant volume and ventricular remodelling when echo windows, eccentric jets, or multiple lesions create uncertainty.^2^ In multivalvular disease, where interacting lesions can distort Doppler assumptions, the EACVI consensus statement underscores multimodality imaging as integral to Heart Team discussion and complex decision-making.^3^

A practical way to state this in guideline-era terms: CMR is often most valuable when the treatment decision depends on chamber consequences, not just valve appearance—especially in mixed or multiple valve disease where ‘severity’ cannot be safely reduced to a single echo measurement.^2,3^

## Prosthetic valves and complications: imaging as a problem-solving stack

As procedural volume rises (surgical and transcatheter), prosthetic valve assessment becomes a major imaging workload—and a major source of misclassification if done with a single modality mindset.

The 2024 ASE guideline on prosthetic valve function explicitly frames echocardiography as foundational but emphasizes the complementary role of CT and CMR, and even Positron Emission Tomography in selected contexts.^4^ The deeper implication for 2025 valve care is that ‘prosthetic evaluation should be pre-planned’—baseline post-implant studies, reproducible follow-up protocols, and early escalation to advanced imaging when haemodynamics and symptoms diverge.

### Interventional imaging: from ‘guidance’ to ‘procedural safety and durability’

The guideline direction—especially for TR—implies more transcatheter procedures in anatomically complex spaces. That means intraprocedural imaging must be treated as a determinant of outcome, not a procedural accessory.

For TR, the guideline recommendation itself is explicitly conditioned on avoiding severe RV dysfunction or pre-capillary PH.^2–10^ This places imaging in a dual role: ‘selection’ (who is still likely to benefit) and ‘guidance’ (how to execute safely within complex right-heart anatomy). In practice, this is where echo (often 3D transesophageal echocardiogram and/or Intracardiac Cardiac Ecocardiography) and CT-derived anatomical understanding must converge into a procedural map that the operator can execute.

## Will artificial intelligence be used as ‘guideline-native’ decision support?

**Figure 1:**
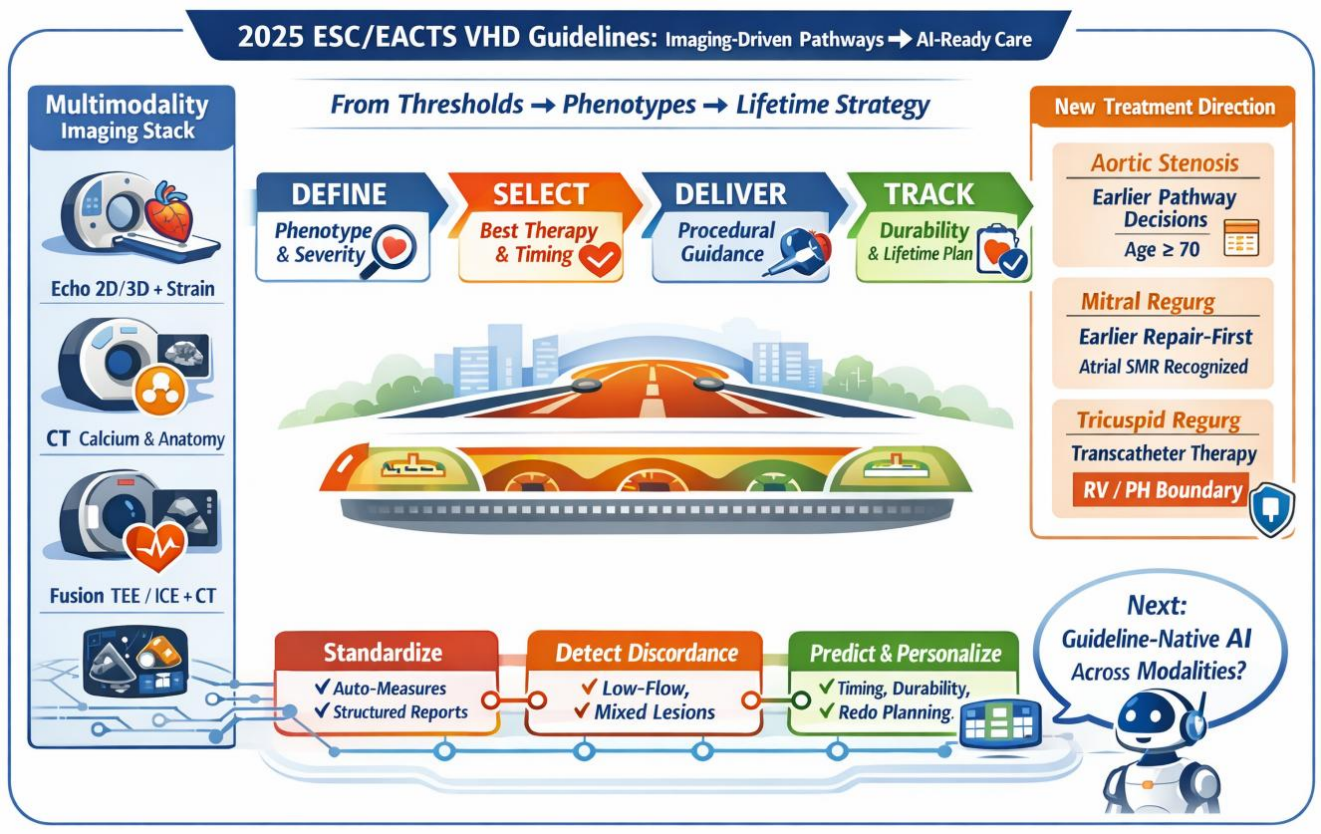
Central illustration on the present and the future of new valvular guidelines.

The most intriguing hint about the future may be embedded in the guideline ecosystem itself: the ESC guideline page explicitly notes that ESC Guidelines are protected by copyright and that use of guideline content to train or develop generative artificial intelligence (AI) models requires a formal licence agreement (*Figure 1*). That statement is not merely legal; it is a signal that AI is expected to intersect with guidelines in clinically meaningful ways—and that governance, attribution, and controlled implementation will matter.

So the open question is not whether AI will enter valve imaging—it already has, through automation and decision support—but what kind of AI we actually need.

Do we want AI that is excellent at segmenting valves and measuring chambers, yet disconnected from guideline logic? Or do we want ‘guideline-native AI’: systems that (i) follow the same integrative pathways (like the AS discordance algorithm), (ii) encode multimodality uncertainty rather than hiding it, and (iii) output structured, auditable statements that a Heart Team can challenge, accept, or override?

Early work suggests feasibility: AI-guided echocardiography acquisition and interpretation can improve workflow and reproducibility, potentially lowering operator dependence—an attractive proposition when guidelines push earlier intervention in asymptomatic disease and expand transcatheter indications where anatomy is decisive.^5^ At the same time, professional societies have emphasized that AI in cardiovascular imaging must be validated, explainable where possible, and integrated into clinical governance rather than treated as a black box.^6^ Even clinician-facing primers increasingly stress that the main value may be ‘standardization and scalability’, not magical new biomarkers.^7^

A plausible ‘next fusion’, then, is not AI replacing multimodality imaging—but AI acting as the connective tissue that makes multimodality practical at scale:

- ‘Standardized acquisition and reporting’ across centres, enabling reliable longitudinal comparisons in lifetime management.
- ‘Automated identification of discordant patterns’ (e.g. AS low-flow phenotypes) that trigger the correct next test rather than an incorrect conclusion.
- ‘Procedural foresight’ in CT planning—anticipating coronary access and redo scenarios as part of the index report, consistent with lifetime framing.
- ‘Risk stratification that respects phenotype’, particularly in TR where benefit hinges on right ventricle/pulmonary hypertension (RV/PH) boundaries.

But that fusion only helps if it remains faithful to the premise of the 2025 guideline direction: patient-centred decisions made by accountable Heart Teams, supported by imaging that is transparent, reproducible, and clinically contextual. The challenge for the next guideline cycle is therefore as much organizational as it is technological: can we build imaging-and-AI workflows that are trustworthy enough to justify earlier intervention and more complex lifetime pathways—without amplifying variability, bias, or overconfidence?

That is the real frontier: not ‘AI in imaging’, but AI that strengthens guideline-based care without eroding clinical accountability.

## Data Availability

No new data were generated or analysed in support of this research.

## References

[1] Praz F, Borger MA, Lanz J, Marin-Cuartas M, Abreu A, Adamo M et al 2025 ESC/EACTS Guidelines for the management of valvular heart disease. Eur Heart J 2025;46:4635–736. 40878295 10.1093/eurheartj/ehaf194

[2] Lancellotti P, Pibarot P, Chambers J, La Canna G, Pepi M, Dulgheru R et al Multi-modality imaging assessment of native valvular regurgitation: an EACVI and ESC Council of Valvular Heart Disease position paper. Eur Heart J Cardiovasc Imaging 2022;23:e171–232. 35292799 10.1093/ehjci/jeab253

[3] Donal E, Unger P, Coisne A, Pibarot P, Magne J, Sitges M et al The role of multi-modality imaging in multiple valvular heart diseases: a clinical consensus statement of the European Association of Cardiovascular Imaging of the European Society of Cardiology. Eur Heart J Cardiovasc Imaging 2025;26:593–608. 39874243 10.1093/ehjci/jeaf026

[4] Zoghbi WA, Jone PN, Chamsi-Pasha MA, Chen T, Collins KA, Desai MY et al Guidelines for the evaluation of prosthetic valve function with cardiovascular imaging: a report from the American Society of Echocardiography developed in collaboration with SCMR and SCCT. J Am Soc Echocardiogr 2024;37:2–63. 38182282 10.1016/j.echo.2023.10.004

[5] Mor-Avi V et al Artificial intelligence–guided echocardiography and its potential clinical impact. Circ Cardiovasc Imaging 2023.

[6] Slart RHJA, Williams MC, Juarez-Orozco LE, Rischpler C, Dweck MR, Glaudemans AWJM et al Position paper of the EACVI and EANM on artificial intelligence applications in multimodality cardiovascular imaging using SPECT/CT, PET/CT, and cardiac CT. Eur J Nucl Med Mol Imaging 2021;48:1399–413. 33864509 10.1007/s00259-021-05341-z PMC8113178

[7] Fortuni F et al Artificial intelligence in cardiovascular imaging: a primer for clinicians. Eur Heart J Imaging Methods Pract 2024;3:qyae034. 10.1093/ehjimp/qyaf149 PMC12687348 41377898

[8] Zancanaro E, Di Mauro M, Chiariello GA, Dohle DS, Kresoja KP, Lin J et al Long-term outcomes of bioprosthetic tricuspid valves: a systematic review of studies published over the last 20 years. Eur Heart J Imaging Methods Pract 2025;3:qyaf097. 41216347 10.1093/ehjimp/qyaf097 PMC12596361

[9] Zancanaro E, Grapsa J, Kresoja KP, Ascione G, Sethi K, Rosch S et al Primary mitral regurgitation, surgery in the transcatheter era: when the neighbourhood becomes noisy: a state-of-art review. Eur Heart J Imaging Methods Pract 2025;3:qyaf041. 40342830 10.1093/ehjimp/qyaf041 PMC12060135

[10] Zancanaro E, Buzzatti N, Guicciardi NA, Denti P, Agricola E, Ancona F et al Real-world outcomes of TMVR-eligible and TMVR-ineligible patients. Eur Heart J Imaging Methods Pract 2025;3:qyaf098. 41058678 10.1093/ehjimp/qyaf098 PMC12499754

